# A Review of the Planthopper Genus *Armacia* Stål (Hemiptera: Fulgoromorpha: Ricaniidae) with Descriptions of Four New Species from Indonesia and Papua New Guinea

**DOI:** 10.1673/031.011.8901

**Published:** 2011-07-17

**Authors:** Cui-Ping Bu, Murray J Fletcher, Ai-Ping Liang

**Affiliations:** ^1^ Key Laboratory of Zoological Systematics and Evolution, Institute of Zoology, Chinese Academy of Sciences, | Beichen West Road, Chaoyang District, Beijing 100101, P.R.China; ^2^ Graduate University of Chinese Academy of Sciences, Beijing 100039, P.R. China; ^3^ Orange Agricultural Institute, Orange, NSW 2800, Australia

**Keywords:** biodiversity, distribution, Fulgoroidea, taxonomy

## Abstract

The genus *Armacia* Stål (Hemiptera: Fulgoromorpha: Ricaniidae) is reviewed taxonomically. Four new species of the genus are described and illustrated from West-Pacific region: *A. madangensis* **sp. nov.** (Papua New Guinea), *A. rubilimba* **sp. nov.** (Indonesia), *A. spinae* **sp. nov.** (Indonesia) and *A. vigorata* **sp. nov.** (Indonesia), *A. albipes* (Walker 1868), *A. clara* (Stål 1859), *A. divisura* (Walker 1868), *A. fusca* Melichar 1898, *A. hyalinata* (Donovan 1805), *A. latipennis* (Walker 1868), *A. nigrifrons* (Walker 1858), *A. simaethis* Fennah 1956, and *A. spatiosa* (Walker 1868) are redescribed and illustrated. A checklist of all known species and a diagnosis of the genus are provided. A key to all species in the genus is provided.

## Introduction

The planthopper family, Ricaniidae, was established by Melichar in 1923 and is one of the larger of the 21 Fulgoroidea families currently recognized, including about 48 genera and over 450 described species (Dlabola 1980; Melichar 1898a, b, 1923; Metcalf 1955; Fennah 1968, 1969, 1971; Williams and Fennah 1980; Yang 1989; Shcherbakov 2006; Fletcher 2008; Bu et al. 2010). Members of the group are distributed primarily around the tropics with 26 genera in the Afrotropical realm, 17 genera in the Australasian realm, 11 genera in the Indo-Malayan realm, and 9 genera in Oceania (Metcalf 1955; Miklos 1975). To date, five genera in the Ricaniidae are endemic to the Australasian region, e.g. *Armacia* Stål, *Epithalamium* Kirkaldy, *Hajar* Kirkaldy, *Motua* Distant, and *Motumotua* Distant. The genus *Armacia* with 13 described species is the largest endemic Australasian planthopper genus in the Ricaniidae. The last comprehensive review of *Armacia* was by Melichar (1898a, 1923). Fletcher (2008) only recorded *A. hyalinata* (Donovan) from Australia and did not treat 12 other species, primarily from Indonesia, Papua, and the Solomon Islands. However, the ricaniid faunas of Indonesia and Papua New Guinea remain inadequately studied and there is still much basic taxonomic work to be done on the group in these regions.

The genus *Armacia* was established by Stål (1862) for *Ricania clara* Stål 1859 from Pouynipet Island, Micronesia. Melichar (1898a) described *Armacia exacta* from New Guinea and *A. fusca* from Buru Island and moved eight species from *Ricania* to the genus. Distant (1909) transferred *Alisca latipennis* (Walker) to *Armacia* and synonymised *Armacia basigera* (Walker 1868) with *A. consobrina* (Walker 1868). Distant (1911) described *A. atrofascialis* from the Solomon Islands. Melichar (1923) transferred *Ricania cribrata* Walker (Figure 69) to *Armacia;* Metcalf (1955) moved it from *Armacia* to *Motua.* Fennah (1956) described *A. simaethis* from the Western Caroline Islands. It should be noted that Melichar (1898a) and Metcalf (1955) incorrectly dated Walker (1868) as Walker (1870). The genus currently contains 13 known species and has a wide distribution in the West-Pacific area (Melichar 1898a, b, 1923; Metcalf 1950, 1955; Fennah 1956, 1971; Fletcher 2008).

While sorting and identifying Ricaniidae from material in the Bernice P. Bishop Museum, Honolulu, Hawaii, USA and elsewhere, four new species of *Armacia* from Indonesia and Papua New Guinea were found. In this paper, the genus *Armacia* and nine previously described species are redescribed and illustrated, in addition to the four new described species. A key is given for the separation of the known species in the genus. A checklist of all known species in the genus is also provided.

## Materials and Methods

The specimens studied in the course of this work are deposited in the following institutions, abbreviated in the text as follows:

BPBM: Bernice Pauahi Bishop Museum, Honolulu, Hawaii, USA;

CAS: California Academy of Sciences, San Francisco, California, USA;

NCSU: Department of Entomology Insect Collection, North Carolina State University, Raleigh, NC, USA;

NRS: Naturhistoriska Riksmuseet, Stockholm, Sweden;

USNM: National Museum of Natural History, Washington DC, USA.

Specimens used for dissection were cleared in 10% KOH at room temperature for ca. 12 hours, rinsed in distilled H_2_O, then transferred to glycerol for examination. Morphological characters were observed with a Zeiss (Stemi SV 11) optical stereomicroscope and were illustrated with the aid of a drawing tube attached to the microscope. Measurements were made with the aid of an eyepiece micrometer.

The morphological terminology followed is that of Bu et al. (2010).

### Taxonomy

**Checklist of the species *of Armacia* Stål**
*Armacia* Stål 1862
*albipes* (Walker 1868). Indonesia (Bacan, Sula Island).
*atrofascialis* Distant 1911. Solomon Islands.
*basigera* (Walker 1868). Indonesia (Amboina, Bacan, Halmahera, Maluku, W. Papua).
*clara* (Stål 1859). Micronesia (Caroline Islands, Ponape Island, Pouynipet Island, Truk Islands), Palau (Angaur Island, Babelthuap Island, Korror Island, Palau Islands).
*colligata* (Walker 1868). Indonesia (Ceram).
*divisura* (Walker 1868). Indonesia (Halmahera, Kai Islands, West Papua).
*exacta* Melichar 1898. Indonesia (West Papua).
*fusca* Melichar 1898. Indonesia (Buru Island).
*hyalinata* (Donovan 1805). Australia (Dorre Island, Queensland, New South Wales?), India?, Indonesia (Amboina, Buru, West Papua, Maluku, Ternate), Papua New Guinea (Offak), Solomon Islands.
*latipennis* (Walker 1868). Australia, Indonesia (Bacan, Sula Island).
*madangensis* **sp. nov.** Papua New Guinea (Madang Province).
*nigrifrons* (Walker 1858). Indonesia (Bacan, Indian Archipelago, Maluku, Sulawesi).
*rubilimba* **sp. nov.** Indonesia (Halmahera Island).
*simaethis* Fennah 1956. Palau (Western Caroline Island).
*spatiosa* (Walker 1868). Indonesia (Misool, West Papua).
*spinae* **sp. nov.** Indonesia (Halmahera Island).
*vigor ata* **sp. nov.** Indonesia (Sulawesi).

**Figures 1–8.**
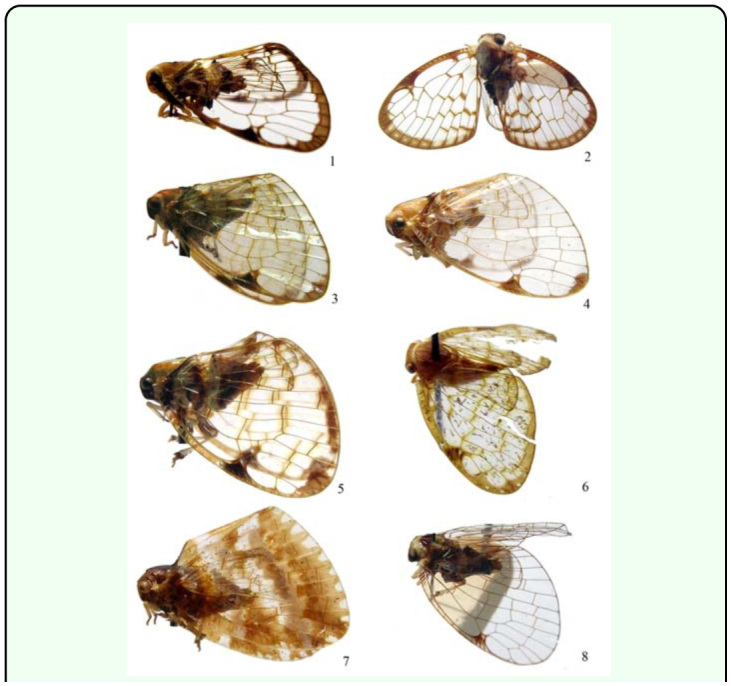
Lateral habitus of *Armacia* species. l. *A. clara* (Stål, 1859), ♂, Micronesia. 2. same, ♀, Palau. 3. *A. hyalinata* (Donovan 1805), ♂, Solomon Islands. 4. same, ♀, Solomon Islands. 5. *A. madangensis* **sp. nov.,** ♂, holotype, Papua New Guinea. 6. *A. rubilimba* **sp. nov.,** ♂, holotype, Indonesia. 7. *A. Spinae* **sp. nov.,** ♂, holotype, Indonesia. 8. *A. vigorata* **sp. nov.,** ♂, holotype, Indonesia. High quality figures are available online.

**Figures 9–17.**
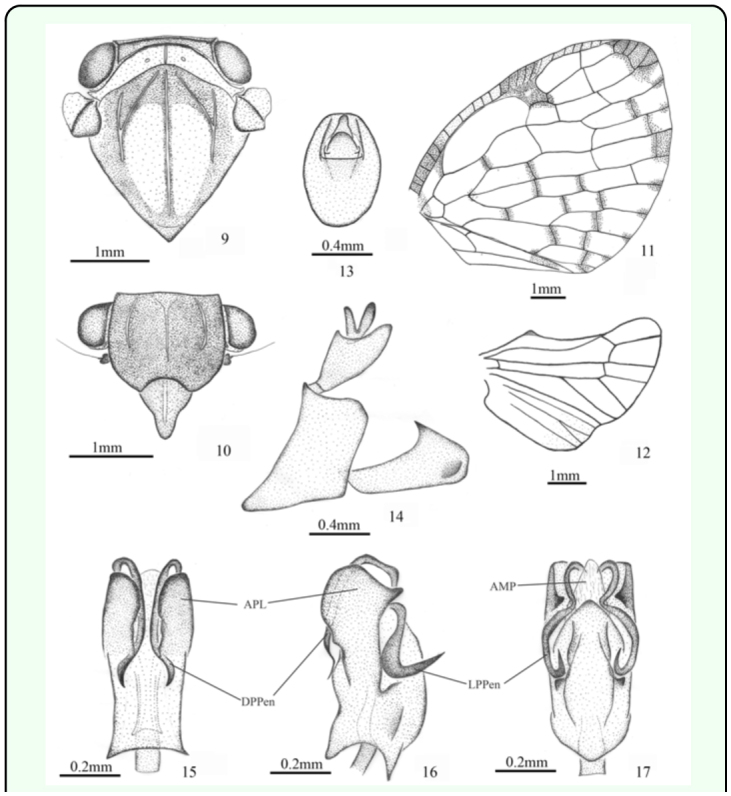
*Armacia madangensis* **sp. nov.,** ♂, holotype. 9. head, pronotum, and mesonotum (dorsal view). 10. head (ventral view). 11. forewing. 12. hindwing. 13. anal tube (dorsal view). 14. genitalia (lateral view). 15. aedeagus (dorsal view). 16. aedeagus (lateral view). 17. aedeagus (ventral view). Abbreviations: **AMP,** apical membranous process of penis; **APL,** apical papillose lobe of penis; **DPPen,** dorsal process of penis; **LPPen,** lateral process of penis. High quality figures are available online.

**Figures 18–26.**
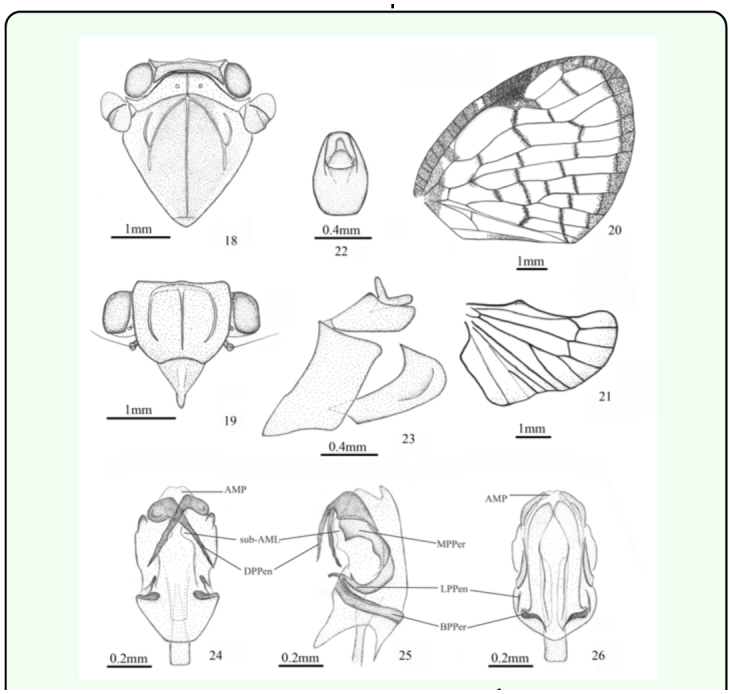
*Armacia rubilimba* **sp. nov.,** ♂, holotype. 18. head, pronotum, and mesonotum (dorsal view). 19. head (ventral view). 20. forewing. 21. hindwing. 22. anal tube (dorsal view). 23. genitalia (lateral view). 24. aedeagus (dorsal view). 25. aedeagus (lateral view). 26. aedeagus (ventral view). Abbreviations: **AMP,** apical membranous process of penis; **sub-AML,** subapical membranous lobe of penis; **DPPen,** dorsal process of penis; **LPPen,** lateral process of penis; **MPPer,** middle process of periandrium; **BPPer,** basal process of periandrium. High quality figures are available online.

**Figures 27–35.**
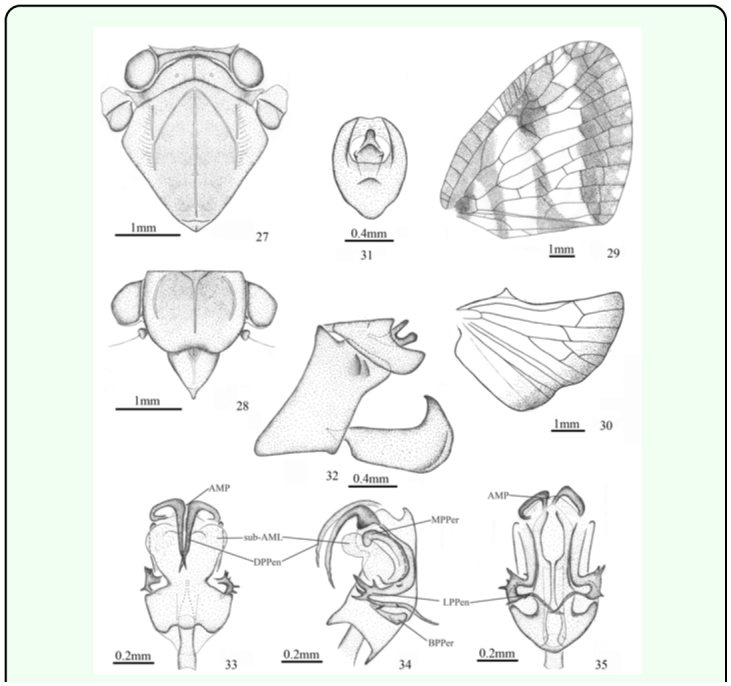
*Armacia Spinae* **sp. nov.,** ♂, holotype. 27. head, pronotum, and mesonotum (dorsal view). 28. head (ventral view). 29. forewing. 30. hindwing. 31. anal tube (dorsal view). 32. genitalia (lateral view). 33. aedeagus (dorsal view). 34. aedeagus (lateral view). 35. aedeagus (ventral view). Abbreviations: **AMP,** apical membranous process of penis; **sub-AML,** subapical membranous lobe of penis; **DPPen,** dorsal process of penis; **LPPen,** lateral process of penis; **MPPer,** middle process of periandrium; **BPPer,** basal process of periandrium. High quality figures are available online.

**Figures 36–44.**
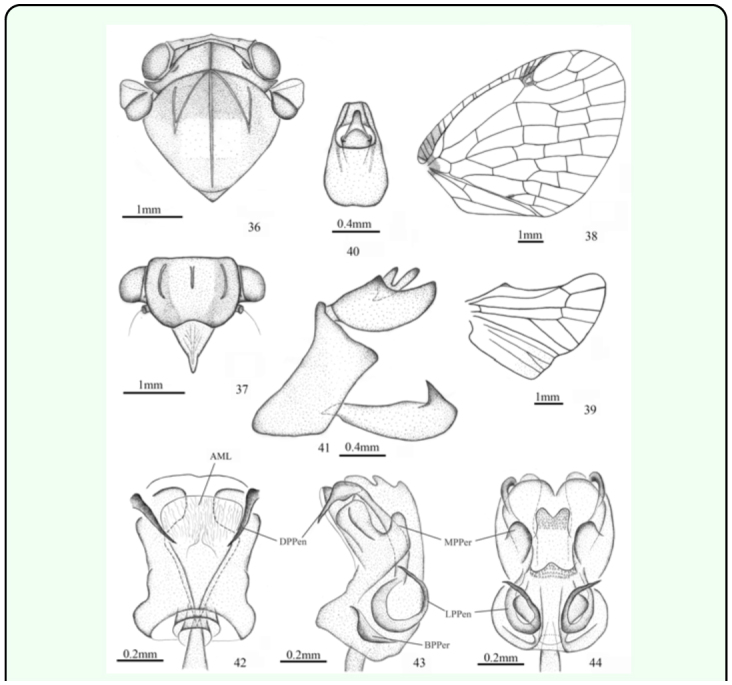
*Armacia vigorata* **sp. nov.,** ♂, holotype. 36. head, pronotum, and mesonotum (dorsal view). 37. head (ventral view). 38. forewing. 39. hindwing. 40. anal tube (dorsal view). 41. genitalia (lateral view). 42. aedeagus (dorsal view). 43. aedeagus (lateral view). 44. aedeagus (ventral view). Abbreviations: **AML,** apical membranous lobe of penis; **DPPen,** dorsal process of penis; **LPPen,** lateral process of penis; **MPPer,** middle process of periandrium; **BPPer,** basal process of periandrium. High quality figures are available online.

**Figures 45–54.**
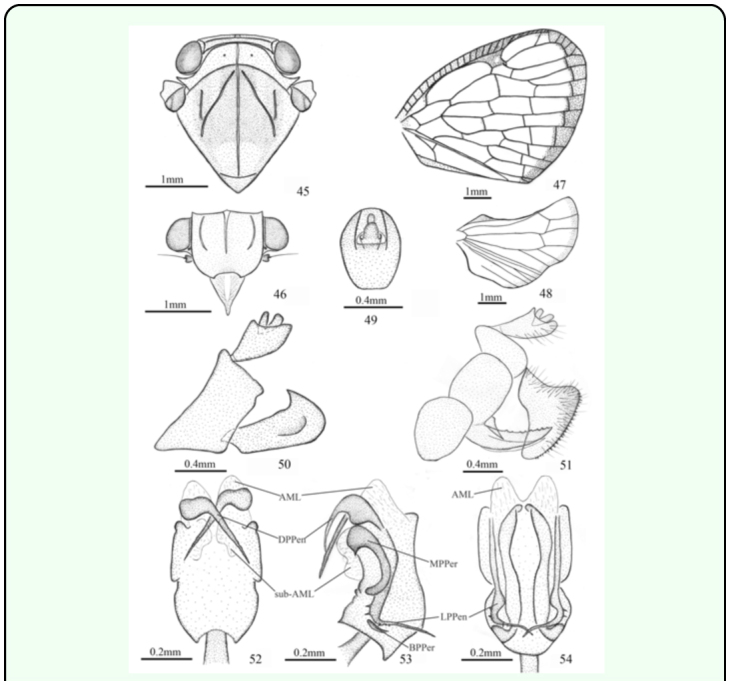
*Armacia clara* (Stål 1859). 45. head (♂), pronotum, and mesonotum (dorsal view). 46. head (♂) (ventral view). 47. forewing (♂). 48. hindwing (♂)*.* 49. anal tube (♂) (dorsal view). 50. genitalia (♂) (lateral view). 51. genitalia (♀) (lateral view). 52. aedeagus (dorsal view). 53. aedeagus (lateral view). 54. aedeagus (ventral view). Abbreviations: **AML,** apical membranous lobe of penis; **sub-AML,** subapical membranous lobe of penis; **DPPen,** dorsal process of penis; **LPPen,** lateral process of penis; **MPPer,** middle process of periandrium; **BPPer,** basal process of periandrium. High quality figures are available online.

**Figures 55–64.**
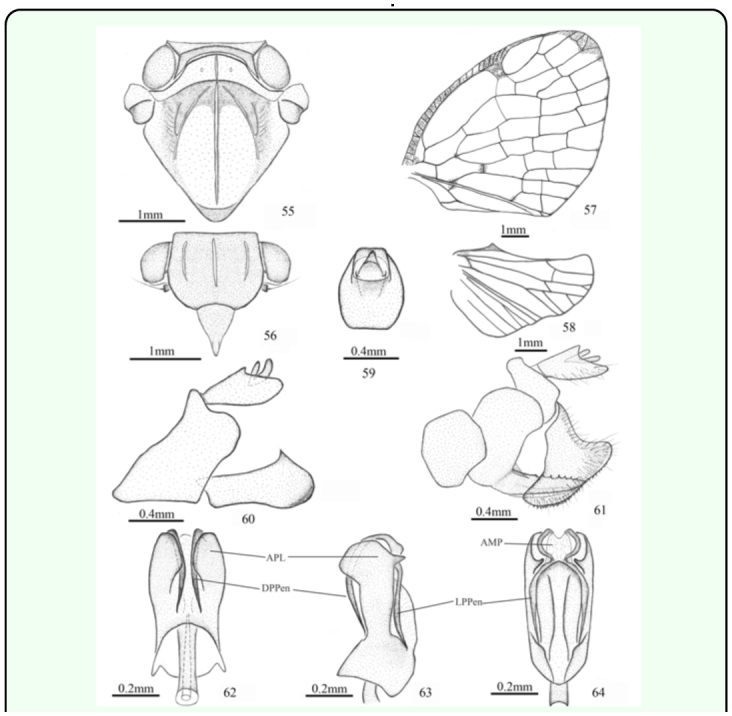
*Armacia hyalinata* (Donovan 1805). 55. head (♂), pronotum, and mesonotum (dorsal view). 56. head (♂) (ventral view). 57. forewing (♂). 58. hindwing (♂). 59. anal tube (♂) (dorsal view). 60. genitalia (♂) (lateral view). 61. genitalia (♀) (lateral view). 62. aedeagus (dorsal view). 63. aedeagus (lateral view). 64. aedeagus (ventral view). Abbreviations: **AMP,** apical membranous process of penis; **APL,** apical papillose lobe of penis; **DPPen,** dorsal process of penis; **LPPen,** lateral process of penis. High quality figures are available online.

**Figures 65–67.**
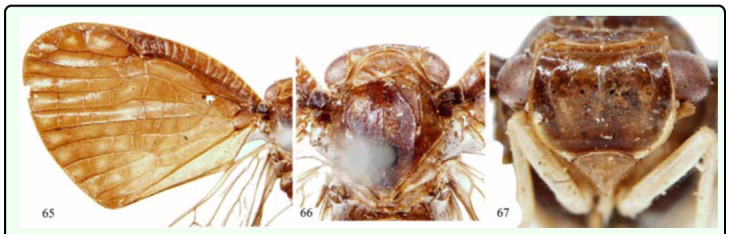
*Armacia fusca* Melichar 1898, ♂, syntype. 65. forewing. 66. head, pronotum, and mesonotum (dorsal view). 67. head (ventral view). High quality figures are available online.

**Figures 68–75.**
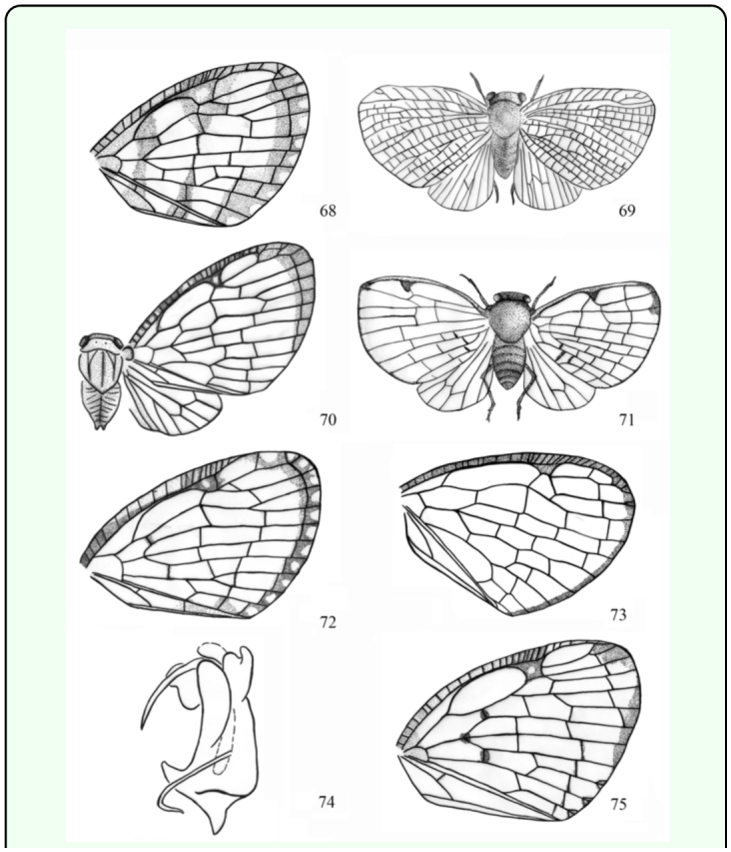
*A. albipes* (Walker 1868), wing. 69. *M. cribrata* (Walker 1868), wing. 70. *A. divisura* (Walker 1868), wing. 71. *A. latipennis* (Walker 1868), wing. 72. *A. nigrifrons* (Walker 1858), wing. 73. *A. simaethis* Fennah 1956, wing. 74. *A. simaethis* Fennah 1956, aedeagus. 75. *A. spatiosa* (Walker 1868), wing. Notes: Figures (69, 71) quoted from Walker (1868), Figures (68, 70, 72, 75) quoted from Melichar (1898a), Figures (73–74) quoted from Fennah (1956). High quality figures are available online.

**Genus *Armacia* Stål 1862**
*Armacia* Stål 1862: 70, 1870: 768; Melichar 1898a: 286, 1923: 145; Metcalf 1950: 65; Fennah 1956: 205. Type species: *Ricania clara* Stål 1859, by original designation.
***Description.*** General colour ochraceous or fuscous. Vertex and most part of frons usually brown or dark brown. Pronotum pallid, sometimes greenish. Legs pale yellow. Forewing and hindwing (Figures 1–8, 11–12, 20–21, 29–30, 38–39, 47–48, 57–58, 65, 68, 70–73, 75) hyaline, more or less fuliginous; veins brown; stigma fuscous, nearly opaque.
  Head (Figures 1–10, 18–19, 27–28, 36–37, 45–46, 55–56, 66–67) large. Vertex (Figures 9, 18, 27, 36, 45, 55, 66) broad and short, distinctly separated from the frons by a transverse carina. Frons (Figures 10, 19, 28, 37, 46, 56, 67) oblique, broader than long, with central, sublateral and lateral carinae; clypeus (Figures 10, 19, 28, 37, 46, 56, 67) narrower than frons, shallowly inserted. Rostrum with subapical segment just surpassing meso-trochanters, apical segment attaining post-trochanters.
  Pronotum (Figures 9, 18, 27, 36, 45, 55, 66) narrow, with a central carina. Mesonotum (Figures 9, 18, 27, 36, 45, 55, 66) large, triangular and convex, with 3 carinae: central carina straight; lateral carinae inwardly and anteriorly curved, converging closely together on anterior margin, each bifurcating outwardly near middle in a straight longitudinal carina. Forewing (Figures 1–8, 11, 20, 29, 38, 47, 57, 65, 68, 70–73, 75) elongate-triangular, with sparse longitudinal veins and quadrate cells; Sc narrowly separated from the costal border with several distinct cross veins before the nodal cell; three veins emanating from basal cell, R branched near base with outer branch running very close to Sc, but inner branch well separated from Sc, M with three branches arising close to the basal cell, Cu1 with three branches just before the apical margin; claval veins united just beyond apex of scutellum, clavus with only one cross vein connecting 1A to claval suture; apical margin longer than claval suture. Hindwing (Figures 12, 21, 30, 39, 48, 58) small, with two cross veins beyond middle and several longitudinal veins forked near apex. Legs moderately long; hind tibiae with 2 lateral black-tipped spines and 6 apical black-tipped spines.
  Male genitalia: pygofer (Figures 14, 23, 32, 41, 50, 60) narrow and high in lateral view. Anal tube (Figures 13, 22, 31, 40, 49, 59) oval or trapeziform, small in dorsal view, short and broad in lateral view. Anal styles relatively short and small, bilobed. Genital styles (Figures 14, 23, 32, 41, 50, 60) symmetrical, relatively elongate and narrow, with apical process angulate or acute at tip. Aedeagus (Figures 15–17, 24–26, 33–35, 42–44, 52–54, 62–64, 74) stout, nearly straight, mostly sclerotised, symmetrical, periandrium well-developed, surrounding penis, distally attached to penis, with pair of cephalad directed dorsal processes at apex, and pair of lateral processes near base.
  Female genitalia (Figures 51, 61) symmetrical. Gonopophyses VIII sawlike, strongly sclerotised with teeth on dorsal margin; gonoplac triangular with many teeth extending along ventral margin.
***Biology.*** As with many ricaniid planthopper species, no biological data are currently available for species of *Armacia.*
***Remarks.*** Species of *Armacia* can be distinguished from other Ricaniidae by the combination of the following diagnostic characters: forewing vitreous with sparse longitudinal veins and quadrate cells; three veins emanating from basal cell; R branched near base with R1 running very close to Sc, but Rs well separated from Sc.
Species of *Armacia* are similar to those of *Alisca* Stål and *Plestia* Stål, which were originally described as subgenera of the genus *Armacia* by Stål (1871). *Alisca* and *Plestia* were elevated to genus by Melichar (1898a) and can be separated from *Armacia* by the forewing having four veins emanating from the basal cell rather than three as in *Armacia.* In addition, the frons of *Plestia* species is transverse-oval, broader than in species of *Armacia.*
***Distribution.*** Australia, India?, Indonesia, Micronesia, Palau, Papua New Guinea, Solomon Islands.

**Key to the species of genus *Armacia***
1 Fore wing pitch brown, R1 running close to Sc but separated from Sc (Figure 65) *A. fusca* Melichar
Forewing vitreous, R1 running very close to Sc, nearly merged (Figures 1–8, 11, 20, 29, 38, 47, 57, 68, 70–73, 75) 2
2 Forewing with three brown transverse bands (Figures 7, 29, 68) 3
Forewing without brown transverse bands (Figures 1–6, 8, 11, 20, 38, 47, 57, 70– 73, 75) 4
3 Forewing with some cross veins near subapical line, subapical line complete (Figures 7, 29) *A. spinae* **sp. nov.**

Forewing without any cross veins near subapical line, subapical line incomplete (Figure 68) *A. albipes* (Walker)
4 Forewing with longitudinal veins forked near apical margin (Figure 75) 5
Forewing with longitudinal veins unforked near apical margin (Figures 1–6, 8, 11, 20, 38, 47, 57, 70, 71, 72, 73) 6
5 Subapical line of fore wing shorter than apical margin (1:3), not parallel with apical margin (Figure 75) *A. spatiosa* (Walker)
Subapical line of fore wing slightly shorter than apical margin, parallel to apical margin *A. exacta* Melichar
6 Forewing with costal margin brown, apical margin brown or hyaline (Figures 1–6, 11, 20, 47, 57, 70–73) 7
Forewing with costal and apical margins lacking brown (Figures 8, 38) *A. vigorata* **sp. nov.**

7 Apical margin of forewing with a brown fascia (Figures 1, 2, 6, 20, 47, 70, 72, 73) 8
Apical margin of forewing without a brown fascia (Figures 3–5, 11, 57, 71) 12
8 Apical margin of forewing with brown fascia complete (Figures 1, 2, 6, 20, 47, 73) 9
Apical margin of forewing with brown fascia incomplete (Figures 70, 72) 11
9 Aedeagus with a pair of ventromesad directed lateral processes near base (Figures 52, 53, 54) *A. clara* (Stål)
Aedeagus with a pair of dorsomesad directed lateral processes near base (Figures 24, 25, 26, 74) 10
10 Aedeagus with short lateral processes (Figures 24–26) *A. rubilimba* **sp. nov.**

Aedeagus with long lateral processes (Figure 74) *A. simaethis* Fennah
11 Subapical line of forewing incomplete, apical brown fascia unbroken to the end of the subapical line (Figure 70) *A. divisura* (Walker)
Subapical line of forewing complete, brown fascia interrupted with a row of hyaline spots (Figure 72) *A. nigrifrons* (Walker)
12 Mesonotum ochraceous with two broad, greenish white stripes along median longitudinal carina (Figures 3, 5, 9, 55) 13
Mesonotum without greenish white stripes (Figure 71) 15
13 Frons nearly twice as wide as long, rounded on each side *A. colligata* (Walker)
Frons somewhat wider than long in middle line, slightly curved on each side (Figures 10, 56) 14
14 Apical margin of forewing with scattered brown spots; aedeagus with dorsal processes not parallel, remote at end, and ventral processes strongly decurved (Figures 11, 15–17) *A. madangensis* **sp. nov.**

Apical margin of forewing without brown spots; aedeagus with dorsal processes nearly parallel, and ventral processes nearly straight (Figures 57, 62–64) *A. hyalinata* (Donovan)
15 Apical margin of forewing with a series of small black spots *A. atrofascialis* Distant
Apical margin of forewing without a series of small black spots (Figure 71) 16
16 Forewing with black apical margin (Figure 71) *A. latipennis* (Walker)
Forewing without black apical margin *A. basigera* (Walker)

***Armacia madangensis* sp. nov.  (Figures 5, 9–17)**
***Description.*** ♂ (n=2), BL: 7.5 mm, FWL: 7.0 mm.
  General colour brown to pale green. Vertex mostly brown. Frons fuscous, suffused with brown in middle. Clypeus pale brown, with a narrow pale yellow stripe at middle. Rostrum pallid. Pronotum greenish white. Mesonotum ochraceous with two broad, greenish white stripes along median longitudinal carina. Thorax fuscous ventrally, marked with greenish white. Legs pale; tarsi and tips of tibiae fuscous; post-femora pale brown. Abdomen pale green or brown ventrally, marked with fuscous; brown dorsally, with fuscous latitudinal strips; pygofer pale brown. Forewing vitreous, with most cross veins clouded with brown; pale brown costal margin, shaded with fuscous on stigma and towards tip; basal cell partly fuscous.
  Head (including compound eyes) (Figures 5, 9–10) slightly wider than pronotum. Vertex (Figure 9) wider at anterior margin than long in middle line (15.5:1). Frons (Figure 10) wider at widest part than long in middle line (1.4:1); disc tricarinate, sublateral carinae shorter than central carina. Clypeus (Figure 10) triangular, without central carina.
  Pronotum (Figure 9) wider at widest part than long in middle line (7.8:1), punctuated beside central carina. Mesonotum (Figure 9) tricarinate on disc, with lateral carinas on each side diverging from the middle carina and disunited on the fore border. Wing venation as in Figures 11–12.
  Male genitalia with pygofer (Figure 14) narrow and high, with dorsal posterior margin smoothly produced posteriorly in lateral view. Anal tube (Figure 13) moderately large, shallowly convex at ventral margin, oval in dorsal view, longer than wide at middle (1.4:1). Genital styles (Figure 14) relatively large, broad in lateral aspect, with apical process acute at tip, in profile longer than wide at middle (3.2:1). Aedeagus (Figures 15–17) stout, nearly straight, mostly sclerotised, symmetrical, with pair of rather large, acutely papillose lobes at apex, and two pairs of long processes at each apical angle: dorsal pair directed cephalad, remote at end, reaching to basal three-fifths, ventral pair shorter than dorsal, strongly decurved, directed ventromesad; penis with one triangular membranous process at apex.
***Material examined.*** Holotype ♂, Papau New Guinea: Madang Province, Sapi Forest Reserve (30 km W Madang) 5° 12′ S 145° 30′ E, 10.ii.1987, Norman D. Penny (CAS). Paratype, Papua New Guinea: 1♂, same data as holotype (CAS).
***Etymology.*** This species is named after its distribution in Madang Province, Papua New Guinea.
***Remarks.*** This species is similar to *A. exacta* Melichar 1898 and *A. spatiosa* (Walker 1868) in appearance, but can be distinguished from the latter by its fore wing with longitudinal veins unforked near apical margin and incomplete subapical line (Figure 11). It can be separated from *A. hyalinata* (Donovan 1805) by its clypeus with a narrow pale yellow stripe at middle (Figure 10), anal tube narrower and higher (Figure 13) and aedeagus with strongly decurved processes (Figures 15–17).
***Distribution.*** Papua New Guinea (Madang Province).

***Armacia rubilimba* sp. nov.  (Figures 6, 18–26)**
***Description.*** ♂ (n=2), BL: 6.5 mm, FWL: 7.5 mm.
  General colour brown, marked with fuscous and ochraceous. Vertex and frons mostly brown. Clypeus brown with a narrow pale yellow stripe at middle. Rostrum pale yellow. Pronotum yellowish white with brown anterior apex. Mesonotum fuscous, with brown on each side. Thorax ventrally pale brown. Legs pale brown, tips of tibiae fuscous. Abdomen brown, with pale red stripes. Forewing with mostly cross veins clouded with brown; costal margin and apical margin brown; basal cell partly brown; stigma fuscous, with white hyaline point. Hindwing with brown apical margin.
  Head (including compound eyes) (Figures 6, 18–19) as wide as pronotum. Vertex (Figure 18) wider at anterior margin than long in middle line (7.8:1). Frons (Figure 19) wider at widest part than long in middle line (1.3:1); disc tricarinate, sublateral carinae shorter than central carina. Clypeus (Figure 19) triangular, without central carina.
  Pronotum (Figure 18) wider at widest part than long in middle line (5.7:1), punctuated beside central carina. Mesonotum (Figure 18) tricarinate on disc, with lateral carinas on each side diverging from the middle carina and united on the fore border. Wing venation as in Figures 20–21.
  Male genitalia with pygofer (Figure 23) narrow and high, with dorsal posterior margin angularly produced posteriorly in lateral view. Anal tube (Figure 22) moderately large, slightly convex at ventral margin, oval in dorsal view, longer than wide at middle (1.5:1). Genital styles (Figure 23) relatively large, broad in lateral aspect, with apical process acute at tip, in profile longer than wide at middle (2.7:1). Aedeagus (Figures 24–26) stout and mostly sclerotised, symmetrical, periandrium surrounding penis, distally attached to penis, with pair of cephalad directed dorsal processes at apex, deeply crossed, pair of ear-like processes near middle at two sides, and pair of dorsomesad directed lateral processes near base, covered with one short fine spine and one long fine spine on dorsal and ventral surfaces, pair of striped processes at base; penis with one medially concave membranous process and pair of irregular membranous lobes on upper margin subapically.
***Material examined.*** Holotype ♂, Indonesia: Halmahera Isl., Jailolo Dist., Kampung Pasir Putih, 0° 53′ N, 127° 41′ E, 15–31.i.1981, AC Messer & PM Taylor (USNM). Paratype, Indonesia: 1♂, same data as holotype (USNM).
***Etymology.*** This species is named for its abdomen with pale red stripes.
***Remarks.*** This species is similar to *A. basigera* (Walker 1868), but can be separated from the latter by its vertex (7.8:1) and pronotum (5.7:1), abdomen with pale red stripes (Figure 6). It can be separated from *A. clara* (Stål 1859) by the apex of the wings (Figure 20) and aedeagus with a pair of dorsomesad directed lateral processes near base (Figures 24–26).
***Distribution.*** Indonesia (Halmahera Island).

***Armacia spinae* sp. nov.  (Figures 7, 27–35)**
***Description.*** ♂ (n=1), BL: 8.5 mm, FWL: 8.0 mm.
  General colour fuscous to pale brown. Vertex mostly brown. Frons fuscous, with brown sides. Clypeus brown, with a Y-shaped pale yellow stripe. Rostrum pale brown. Pronotum brown. Mesonotum fuscous. Thorax ventrally fuscous, marked with pale brown. Legs pale, marked with brown; tarsi and tips of tibiae fuscous; post-femora brown. Abdomen yellowish, with yellowish brown bands dorsally; pygofer pale brown. Forewing with three brown bands; basal cell partly fuscous; stigma fuscous, with white hyaline point. Hindwing with brown apical margin.
  Head (including compound eyes) (Figures 7, 27–28) as wide as pronotum. Vertex (Figure 27) wider at anterior margin than long in middle line (11.4:1). Frons (Figure 28) wider at widest part than long in middle line (1.4:1); disc tricarinate, sublateral carinae shorter than central carina. Clypeus (Figure 28) triangular, with short central carina.
  Pronotum (Figure 27) wider at widest part than long in middle line (5.6:1), punctuated beside central carina. Mesonotum (Figure 27) tricarinate on disc, with lateral carinas on each side diverging from the middle carina and united on the fore border. Wing venation as in Figures 29–30.
  Male genitalia with pygofer (Figure 32) narrow and high, with dorsal posterior margin angularly produced posteriorly in lateral view. Anal tube (Figure 31) moderately large, distinctly convex at ventral margin, oval in dorsal view, longer than wide at middle (1.4:1). Genital styles (Figure 32) relatively large, broad in lateral aspect, with apical process acute at tip, in profile longer than wide at middle (2.8:1). Pygofer, anal tube, and genital styles with wrinkle. Aedeagus (Figures 33–35) stout and mostly sclerotised, periandrium surrounding penis, distally attached to penis, with pair of cephalad directed dorsal processes at apex, crossed at end, pair of ear-like processes near middle at two sides, and pair of ventromesad directed lateral processes near base, covered with numerous fine spines on dorsal and ventral surfaces near middle, pair of small triangular processes at base; penis with one medially convex membranous process at apex and pair of oval membranous lobes on upper margin subapically.
***Material examined.*** Holotype ♂ Indonesia: Halmahera Isl., Jailolo Dist., Kampung Pasir Putih 0° 53′ N, 127° 41′ E, 1–14.vii.1981, AC Messer & PM Taylor (USNM).
***Etymology.*** This species is named for its lateral process of aedeagus with numerous fine spines.
***Remarks.*** This species can be distinguished from other known species in *Armacia* by its clypeus with a Y-shaped pale yellow stripe (Figure 28), forewing with three brown bands (Figure 29), and wrinkled pygofer and anal tube (Figure 31–32). It is similar to *A. albipes* (Walker 1868) in appearance, but can be distinguished from the latter by the apices of the fore wing, subapical cells, and many cross veins near subapical line (Figure 29).
***Distribution.*** Indonesia (Halmahera Island).

***Armacia vigorata* sp. nov. (Figures 8, 36–44)**
***Description.*** ♂ (n=1), BL: 9.5 mm, FWL: 8.5 mm.
  General colour fuscous, marked with ochraceous and yellowish green. Vertex mostly brown. Frons fuscous, with pale brown stripe along central carina. Clypeus and rostrum yellowish. Pronotum light greenish yellow. Mesonotum fuscous, with light greenish yellow spot near middle. Thorax ventrally fuscous, marked with pale brown. Legs pale, post-femora with distinct long brown stripes, tarsi and tips of tibiae fuscous; post-femora fuscous. Abdomen fuscous ventrally; dark brown dorsally, with yellowish brown bands; pygofer brown. Forewing with hyaline apical margin; costal margin hyaline, with some cross veins clouded with fuscous at base; basal cell partly fuscous; stigma fuscous, with white hyaline point.
  Head (including compound eyes) (Figures 8, 36–37) slightly wider than pronotum. Vertex (Figure 36) wider at anterior margin than long in middle line (16.5:1), with occipital margin carinate. Frons (Figure 37) wider at widest part than long in middle line (1.6:1); disc tricarinate, sublateral carinae longer than central carina. Clypeus (Figure 37) triangular without central carina.
  Pronotum (Figure 36) wider at widest part than long in middle line (7.5:1), indistinctly punctuated beside central carina. Mesonotum (Figure 36) tricarinate on disc, with lateral carinas on each side diverging from the middle carina and united on the fore border. Wing venation as in Figures 38–39.
  Male genitalia with pygofer (Figure 41) narrow and high, with dorsal posterior margin angularly produced posteriorly in lateral view. Anal tube (Figure 40) moderately large, slightly convex at ventral margin, trapeziform in dorsal view, longer than wide at middle (1.4:1). Genital styles (Figure 41) slender, with apical process acute at tip, in profile longer than wide at middle (3.4:1). Aedeagus (Figures 42–44) short, stouter, mostly sclerotised, symmetrical, periandrium surrounding penis, distally attached to penis, with pair of short cephalad directed dorsal processes at apex, pair of oblong processes near middle at two sides, and pair of long winding lateral processes near base, pair of moderately large triangular processes at base; penis with dorsally rather large, triangular, membranous lobe at apex.
***Material examined.*** Holotype ♂, Indonesia: Celebes I. Lake Lindu, 900m, 1–5.i.1966., R. Straatman Malaise Trap BISHOP (BPBM)
***Etymology.*** This species is named for its very stout aedeagus.
***Remarks.*** This species can be distinguished from other known species in *Armacia* by its pronotum with indistinct punctate spots remote from central carina (Figure 36), wing vitreous without brown margin (Figure 38), anal tube trapeziform in dorsal view (Figure 40), genital styles more slender and aedeagus stouter (Figures 41–44).
***Distribution.*** Indonesia (Sulawesi).

***Armacia clara* (Stål 1859) (Figures 1–2, 45–54)**
*Ricania clara* Stål 1859: 281.
*Armacia clara* (Stål); Melichar 1898a: 287; Metcalf 1950:65; Fennah 1956: 205.
***Redescription.*** ♂ (n=8), BL: 7.0 mm, FWL: 7.0 mm; ♀ (n=2), BL: 8.5 mm, FWL: 8.0 mm.
  General colour brown to fuscous. Pronotum pallid, sometimes tinged with green. Mesonotum ochraceous. Legs paler, tarsi and tips of tibiae fuscous. Costal margin of fore wing with a narrow, brown fascia; apical margin with a broad brown fascia.
  Head (including compound eyes) (Figures 1, 2, 45–46) as wide as pronotum. Vertex (Figure 45) wider at anterior margin than long in middle line (13.6:1). Frons (Figure 46) wider at widest part than long in middle line (1.2:1); disc tricarinate, sublateral carinae slightly shorter than central carina. Clypeus (Figure 46) triangular, without central carina.
  Pronotum (Figure 45) wider at widest part than long in middle line (5.5:1), punctuated beside central carina. Mesonotum (Figure 45) tricarinate on disc, with lateral carinas on each side diverging from middle carina and disunited on fore border. Wing venation as in Figures 47–48.
  Male genitalia with pygofer (Figure 50) narrow and high, with dorsal posterior margin angularly produced posteriorly in lateral view. Anal tube (Figure 49) moderately large, slightly convex at ventral margin, oval in dorsal view, longer than wide at middle (1.3:1). Genital styles (Figure 50) relatively large, broad in lateral aspect, with apical process acute at tip, in profile longer than wide at middle (2.5:1). Aedeagus (Figures 52–54) stout and mostly sclerotised, symmetrical, periandrium surrounding penis, distally attached to penis, with pair of cephalad directed dorsal processes at apex, deeply crossed, pair of ear-like processes near middle at two sides, and pair of ventromesad directed lateral processes near base, covered with two to five fine spines on dorsal and ventral surfaces; penis with one deeply concave membranous lobe at apex and pair of irregular membranous lobes on upper margin subapically.
  Female genitalia (Figure 51) with anal tube relatively small, ventral margin in profile slightly convex; anal style small, bilobed. Gonopophyses VIII (Figure 51) sawlike, strongly sclerotised with about 8 blunt teeth on dorsal margin. Gonoplac (Figure 51) triangular with many teeth extending along ventral margin.
***Type material examined.*** 1♀ (Syntype), Ins Ascens, Kinb, NHRS-HEMI 000000165 (NRS).
***Other material examined.*** 1♀, Palau: Angaur: *Saipan-Kitamura,* 26.ii.1938, Teiso Esaki (NCSU); 1♂*,* Caroline Islands: Truk Island, Moen, 8.xiii.1970, J. E. Tobler & I. Cllr (CAS); 1♂*,* Truk: Tol. I., Mt. Unibot, 2.i.1953, alt. 390m, Pac. Sci. Bd., J. L. Gressitt (CAS); 1♂*,* Truk: Moen I Mt. Teroken, N, 28.xii.1952, Caroline Is., Pac. Sci. Bd., J. L. Gressitt (CAS); 1♂*,* East Caroline Islands, 27.ix.1970, Moen, Truk, M. R. Lundgren, (CAS); 1♂*,* Ponape: Jokaj I. alt. 2m, 29.i.1953, Pacific Sci. Bd. Micronesia Surv, J. L. Gressitt (CAS); 1♂*,* Ponape: Mt. Tamatamansakir, 180m, 18.i.1953, Light trap, Pac. Sci. Bd., J. L. Gressitt (CAS); 2♂♂*,* 1♀, Ponape 6 mi E Colonia, low elev., 12.xii.1976, J.F.G. Clarke, Thelma M Clarke (BPBM).
***Distribution.*** Micronesia (Caroline Islands, Ponape Island, Pouynipet Island, Truk Islands), Palau (Angaur Island, Babelthuap Island, Korror Island, Palau Islands).
***Remarks.*** This species can be distinguished from other known species in *Armacia* by its penis with one large deeply concave membranous lobe at apex (Figures 52–54). *A. clara* includes five subspecies, *A. clara clara* (Stål), *A. clara pallescens* Metcalf, *A. clara trukensis* Fennah, *A. clara namana* Fennah, and *A. clara kusaieana* Fennah, all differentiated by minor variation in colour, particularly of the forewing.

***Armacia hyalinata* (Donovan 1805) (Figures 3–4, 55–64)**
*Cicada hyalinata* Donovan 1805: 2.
*Ricania hyalinata* (Donovan); Guérin-Méneville 1834: 466.
*Ricania donovanii* Spinola 1839: 397, synonymised by Melichar 1898a: 287. *Armacia hyalinata* (Donovan); Melichar 1898a: 287.
*Redescription.* ♂ (n=4), BL: 7.5 mm, FWL: 7.0 mm; ♀ (n=1), BL: 8.5 mm, FWL: 8.0 mm.
  General colour pale brown to fuscous. Vertex mostly pale brown. Frons fuscous. Clypeus pale brown, suffused with brown in middle. Rostrum pallid. Pronotum greenish white. Mesonotum ochraceous with two broad, greenish white stripes along median longitudinal carina. Thorax fuscous ventrally, marked with yellowish white. Legs pale, tarsi and tips of tibiae fuscous; post-femora fuscous. Fore wing with brown costal margin, shaded with fuscous on stigma and towards tip.
  Head (including compound eyes) (Figures 3– 4, 55–56) slightly wider than pronotum. Vertex (Figure 55) wider at anterior margin than long in middle line (11.7:1). Frons (Figure 56) wider at widest part than long in middle line (1.3:1); disc tricarinate, sublateral carinae shorter than central carina. Clypeus (Figure 56) triangular, without central carina.
  Pronotum (Figure 55) wider at widest part than long in middle line (5.1:1), punctuated beside central carina. Mesonotum (Figure 55) tricarinate on disc, with lateral carinas on each side diverging from the middle carina and disunited on the fore border. Wing venation as in Figures 57–58.
  Male genitalia with pygofer (Figure 60) narrow and high, with dorsal posterior margin circularly produced posteriorly in lateral view. Anal tube (Figure 59) moderately large, shallowly convex at ventral margin, oval in dorsal view, longer than wide at middle (1.2:1). Genital styles (Figure 60) relatively large, broad in lateral aspect, with apical process acute at tip, in profile longer than wide at middle (2.4:1). Aedeagus (Figures 62–64) stout, nearly straight, mostly sclerotised, symmetrical, with pair of rather large, acutely papillose lobes at apex, and two pairs of long processes at each apical angle: dorsal pair directed cephalad, reaching to basal three-fifths, ventral pair shorter than dorsal, nearly straight, directed cephalad, nearly paralleled; penis with one sightly concave membranous process at apex.
  Female genitalia (Figure 61) with anal tube relatively small, ventral margin in profile slightly convex; anal style small, bilobed. Gonopophyses VIII (Figure 61) sawlike, strongly sclerotised with about 7 teeth on dorsal margin. Gonoplac (Figure 61) triangular with many teeth extending along ventral margin.
***Material examined.*** 1♀, Solomon Islands: Guadalcanal Koli Point, 30.x. 1944, David G. Hall (BPBM); 1♂*,* Solomon Islands: Guadalcanal Honiara, 0–100m, xii.1971, NLH Krauss (BPBM); 1♂*,* Naval Base Samar, P.I., iv.1945, G. E. Bohart (CAS); 1♂, Solomon IS., Guadalcanal, nr. Tetere, Roront, 24.v.1960, C.W. O'Brien Collector (CAS); 1♂*,* Solomon Is., Vella Lavella Ulo Crater, 10m, 7.xii.1963, Malaise Trap Bishop, P.S. (BPBM).
***Distribution.*** Australia (Dorre Island, Queensland, New South Wales?), India?, Indonesia (Amboina, Buru, West Papua, Maluku, Ternate), Papua New Guinea (Offak), Solomon Islands.
***Remarks.*** This species can be distinguished from other known species in *Armacia* by the characters given in the key. Metcalf (1955) listed Africa as its locality. In fact, it is distributed in Papua New Guinea (Offak) instead of Africa (Walker 1851). Donovan's type material was not found in the Macleay Museum collection (Fletcher 2008).

***Armacia albipes* (Walker 1868) (Figure 68)**
*Ricania albipes* Walker 1868: 154.
*Ricania viridicollis* Walker 1868: 156, synonymised by Melichar 1898a: 288.
*Armacia albipes* (Walker); Melichar 1898a: 288
***Diagnosis.*** ♂, length (excl. tegm.): 5.0 mm; exp. tegm.: 16.0 mm.
  General colour brown to fuscous. Wings hyaline, colourless. Forewing with three brown bands and some hyaline spots along the apical margin.
***Distribution.*** Indonesia (Bacan, Sula Island).

***Armacia divisura* (Walker 1868) (Figure 70)**
*Ricania divisura* Walker 1868: 157.
*Armacia divisura* (Walker); Melichar 1898a: 289.
***Diagnosis.*** ♂*,* length (excl. tegm.): 5.0 mm; exp. tegm.: 19.0 mm.
  General colour testaceous. Frons and clypeus fuscous. Pronotum whitish or whitish green. Leg pale brown. Wings hyaline. Forewing with brown costal margin and apical margin; subapical line incomplete, brown fascia extend to the end of subapical line.
***Distribution.*** Indonesia (Halmahera, Kai Islands, New Guinea).

***Armacia fusca* Melichar 1898  (Figures 65–67)**
*Armacia fusca* Melichar 1898b: 397; Melichar 1898a: 287.
***Diagnosis.*** ♂, length (excl. tegm.): 5.0 mm; exp. tegm.: 16.0 mm.
  General colour fuscous. Forewing opaque, pitch brown; hind wing vitreous. Leg yellowish. Wing venation as in Figure 65.
***Type material examined.*** 1♂ (Syntype), Ins. Baru, Stevens, NHRS-HEMI 000000166 (NRS).
***Distribution.*** Indonesia (Buru Island).

***Armacia latipennis* (Walker 1868)  (Figure 71)**
*Ricania latipennis* Walker 1868: 160.
*Ricania emarginata* Walker 1868: 160, synonymised by Melichar 1923: 146.
*Alisca latipennis* (Walker); Melichar 1898a: 293.
***Diagnosis***, ♂♀, length (excl. tegm.): 6.4–7.4 mm; exp. tegm.: 19.0–21.0 mm.
  General colour testaceous. Vertex very short. Wings vitreous. Forewing broad, with a black marginal line; shaded with blackish near the base, on stigma and towards tip.
***Distribution.*** Australia, Indonesia (Bacan, Sula Island).

***Armacia nigrifrons* (Walker 1858) (Figure 72)**
*Flatoides nigrifrons* Walker 1858: 101.
*Ricania aperta* Walker 1868: 156, synonymised by Melichar 1898a: 228.
*Armacia nigrifrons* (Walker); Melichar 1898a: 288.
***Diagnosis.*** ♂♀, length (excl. tegm.): 5.0–6.0 mm; exp. tegm.: 16.0–21.0 mm.
  General colour fuscous. Vertex very short. Wings vitreous. Forewing shaded with brown near the base of costal membrane and apical margin; brown fascia of apical margin with row of hyaline spots.
***Distribution.*** Indonesia (Bacan, Indian Archipelago, Maluku, Sulawesi).

***Armacia simaethis*Fennah 1956 (Figures 73–74)**
*Armacia simaethis* Fennah, 1956: 209.
***Diagnosis.*** ♂, length (excl. tegm.): 4.0 mm; FWL: 5.9 mm. ♀, length (excl. tegm.): 4.7 mm; FWL: 8.0 mm.
  General colour brown. Forewing vitreous, costal and apical margins narrowly castaneous. Frons wider at widest part than long in middle line (1.25:1). Forewing with subapical line not forming a row for more than half width of fore wing, relatively remote from apical margin (Figure 73). Aedeagus stout, with pair of cephalad directed dorsal processes at apex and pair of dorsomesad directed long lateral processes near base (Figure 74).
***Distribution.*** Palau (Western Caroline Island).

***Armacia spatiosa* (Walker 1870) (Figure 75)**
*Ricania spatiosa* Walker 1870: 157.
*Armacia spatiosa* (Walker); Melichar 1898a: 289.
***Diagnosis.*** ♂♀, length (excl. tegm.): 5.0–5.3 mm; exp. tegm.: 16.0–19.0 mm.
  General colour testaceous. Pronotum and mesonotum greenish white. Wings vitreous. Forewing with brown stigma and apical angle and a narrow, brown fascia at the middle of apical margin (Figure 75).
***Distribution.*** Indonesia (Misool, West Papua).

## Discussion

The taxonomy of Ricaniidae was traditionally based mostly on the morphology of the head and wing, in particular the shape of frons and wing veins. The present study on the genus *Armacia,* confirms that these characters are the prime diagnostic structures. At the same time, interspecific variation of the aedeagus was added.

In biogeography, the genus *Armacia,* as reviewed here, comprises 17 species and is endemic to the Australasian region. All species of the genus *Armacia* are distributed to the east of the Wallace line in eastern Indonesia. The genus *Armacia* is closely related to *Alisca* and *Plestia.* The genus *Alisca* with 3 species is distributed mainly in the Philippines, which is located to the northwest of Wallace's line, but east of Huxley's extension. The genus *Plestia* with 28 species is distributed mainly in the South Pacific Region (Fiji, New Caledonia, Samoa, Tonga, Vanuatu).

## Abbreviations

**BL**,: body length (from apex of cephalic process to tip of fore wing);
**FWL**,: forewing length
